# Deep transverse friction massage in the management of adhesive capsulitis: A systematic review

**DOI:** 10.12669/pjms.40.3.7218

**Published:** 2024

**Authors:** Shahid Khan, Aatik Arsh, Sargand Khan, Shahid Ali

**Affiliations:** 1 Shahid Khan, DPT, MSPT Institute of Physical Medicine and Rehabilitation, Khyber Medical University, Peshawar, Pakistan; 2 Aatik Arsh, DPT, MSPT, PhD Institute of Physical Medicine and Rehabilitation, Khyber Medical University, Peshawar, Pakistan; 3 Sargand Khan, DPT, MSPT Institute of Physical Medicine and Rehabilitation, Khyber Medical University, Peshawar, Pakistan; 4 Shahid Ali DPT, MSPT Institute of Physical Medicine and Rehabilitation, Khyber Medical University, Peshawar, Pakistan

**Keywords:** Adhesive capsulitis, Manual Therapy, Pain, Physical Therapy, Rehabilitation, Shoulder

## Abstract

**Objectives:**

To review published clinical trials which assessed the effects of deep transverse friction massage on pain and range of motion in patients with adhesive capsulitis.

**Methods:**

A systematic review was conducted according to PRISMA guidelines. Literature search was performed in MEDLINE, AMED, EMBASE, HMIC, CINAHL, PEDRO, and SPORTDiscus. Two independent reviewers performed screening of the articles retrieved from different databases. Clinical trials published in English language from the earliest record to March 2022 that reported effects of deep transverse friction massage/Cyriax’s friction massage on pain and/or range of motion in patients with diagnosis of adhesive capsulitis were included. The Critical Appraisal Skills Programme was used for quality assessment of the included studies.

**Results:**

A total of six studies reporting on 226 adhesive capsulitis patients were included in the systematic review. All the six studies were randomized controlled clinical trials. On the Critical Appraisal Skills Programme tool, four of the six studies had a score of 8/11, while the other two studies received a score of 7/11 and 6/11. Out of these six trials, four reported that pain was significantly (P<0.05) improved in the deep transverse friction massage group as compared to the control group. Regarding range of motion outcome, five studies showed that range of motion was significantly (P<0.05) improved in the deep transverse friction massage group while only one study showed non-significant results.

**Conclusion:**

It can be concluded that deep transverse friction massage significantly relieves pain and improves the range of motion in individuals with adhesive capsulitis.

## INTRODUCTION

Adhesive capsulitis, often termed as frozen shoulder, is a common musculoskeletal disorder problem characterized by scapulohumeral pain and limited range of motion (ROM).^1^ Patients suffering from adhesive capsulitis experience a painful restriction in both active and passive movements, most prominently in external rotation.^2^ It is estimated that adhesive capsulitis affects 2% of the general population and has a prevalence rate of 2.4 per 1000 persons per year.^3^,^4^ A wide variety of conservative and non-conservative treatment strategies are used for the management of adhesive capsulitis.^1^ Conservative treatments that are commonly used are muscle stretching and strengthening, mobilization techniques, massage therapy, ultrasound and diathermy.^5^,^6^

Deep transverse friction massage (DTFM), also known as Cyriax friction massage, is a form of massage technique that focuses on the body’s deep tissues.^7^ It is administered to maintain the ROM present inside the ligament, tendon, and muscle’s soft tissue structures and prevent the formation of scars.^8^ DTFM uses strong movements of the hand to physically manipulate the affected tissues. The massage is deep and should be delivered in transverse direction to the particular tissue affected, as opposed to superficial massage applied longitudinally parallel to the vessels, which promotes circulation and fluid return.^9^,^10^ The therapist uses fingertips, pads, knuckles or even elbows. For further pressure one hand reinforced on the other technique can be used. DTFM results in traumatic hyperemia which results in removal of pain aggravating metabolites and movement of the affected structure releasing the adhesions and scar tissue.^11^ DTFM restores muscle mobility in the same manner as mobilization does, while the pressure exerted is within the patient’s tolerance.^12^,^13^

Evidence suggests that DTFM can help reduce pain and improve ROM, nevertheless there is scarcity of high-quality evidence to support the use of DTFM in patients with diagnosis of adhesive capsulitis. Therefore, current study was designed to review published clinical trials which assessed effects of DTFM on pain and ROM in patients with adhesive capsulitis.

## METHODS

A systematic review was conducted according to PRISMA guidelines. Literature search was performed in MEDLINE, AMED, EMBASE, HMIC, CINAHL, PEDRO and SPORTDiscus using search terms “Adhesive Capsulitis” “Frozen Shoulder” “Deep transverse friction massage” “Cyriax’s friction massage” “Pain” and “Range of motion”. Boolean operators and truncations were used where appropriate.

In accordance with PRIMSA guidelines, complete search strategy for one database (CINAHL) is presented in Table-I. General search was performed in Google scholar to find any additional articles. Furthermore, reference list of the retrieved articles from different databases were also checked to locate relevant studies.

**Table-I:** Search strategy used for CINAHL database.

Search	Concept	Search String
S1	Adhesive capsulitis	(MH “Adhesive Capsulitis” OR “Frozen shoulder”) OR (MJ “Adhesive Capsulitis” OR “frozen shoulder”)
S2		(TI Adhesive Capsulitis OR frozen shoulder) OR (AB Adhesive Capsulitis OR frozen shoulder)
S3		(Adhesive Capsulitis OR frozen shoulder OR shoulder contracture syndrome)
S4		S1 OR S2 OR S3
S5	Deep transverse friction massage	(MH “Deep Transverse Friction Massage”) OR (MJ “Deep Transverse Friction Massage”)
S6		(TI Deep Transverse Friction Massage OR (AB Deep Transverse Friction Massage)
S7		(Deep Transverse Friction Massage OR Cross friction massage OR Cyriax massage)
S8		S5 OR S6 OR S7
S9	Control	(MH “Usual Care” OR “standard care” OR “usual treatment”) OR (MJ “Usual Care” OR “standard care” OR “usual treatment”)
S10		(TI Usual Care OR standard care OR usual treatment) OR (AB Usual Care OR standard care OR usual treatment)
S11		Physiotherapy OR Physical therapy OR Rehabilitation
S12		S9 OR S10 OR S11
S13	Outcomes	(Pain OR NRS pain scale OR VAS) OR (Range of motion OR ROM OR range of movement OR Goniometry)
S14	Final Search	S4 AND S8 AND S12 AND S13

**Table-II:** Summary of the included studies

Author	Participants information	Arm	Intervention	Pain				Study Conclusions

				Baseline	Post-intervention	Baseline	Post-intervention	
Sonkusale et al. 2016^12^	60 male and female participants Age=40-60 years	Experimental	DTFM and PNF 6 sessions for 2 weeks	3.567 ±1.569	0.7667± 0.8976	ER 28.667 ± 17.748	47.333± 18.503	In comparison to control group, pain and ROM was significantly (P<0.01) improved in experimental group
		Control	Only PNF 6 sessions for 2 weeks	2.633± 1.273	2 ± 1.339	ER 28.113 ± 16.111	37.7 ± 15.75	
Guler-Uysal & Kozanogl 2004 16	40 participants (12 male and 28 female) Age=40-85 years	Experimental	DTFM, 1 hour session, 3 days per week for 2 weeks	30.6 ± 22.9	15.2 ± 18.5	FLX 128.6 ± 18.6 ABD 114.8 ± 22.3 IR 48.2 ± 11.9 ER 40.8 ± 11.7	155.5 ± 14.2 157.7 ± 21.6 66.7 ± 10.0 74.4 ± 14.2	In comparison to control group, pain and ROM was significantly (P <0.05) improved in experimental group
		Control	Hot pack and diathermy for 20 mints followed by stretching and pendulum exercises for 2 weeks	37.1 ± 24.0	21.2 ± 17.9	FLX 125.8 ± 24.9 ABD 116.0 ± 25.6 IR 42.7 ± 13.7 ER 36.3 ± 16.5	146.4 ± 22.7 145.3 ± 28.5 56.1 ± 14.7 52.8 ± 24.3	
Mogahed et al., 2020^20^	40 female participants Age=35-65 years	Experimental	DTFM and scapular PNF 15 mints session, 3 sessions per week for 2 months	69.2±8.7	52.1±10.9	FLX 65.5±6.46 ABD 68.5±17.85	173.75±6. 04 166±10.2	In comparison to control group, pain and ROM was significantly (P<0.01) improved in experimental group
		Control	Traditional shoulder exercises, 3 sessions per week for 2 months	68±10.2	55.6±0.1	FLX 63.5±12.57 ABD 70.5±10.87	136.25±9. 71 124±14.29	
Sah et al. 2017 17	30 participants (19 male & 11 female) Age=46-60 years	Experimental	DTFM in the form of Cyriax manipulation and conventional physical therapy, 45-minute session, 3 times per week for 2 weeks	64.60±8.7	23.73±6.52	AROM 83.33±17.55 PROM 90.00±17.55	AROM 98.87±20.64 PROM 105.13±20.24	There was no significant difference (P>0.05) between the experimental and control group about pain and ROM.
		Control	Gongs mobilization and conventional physical therapy, 45-minute session, 3 times per week for 2 weeks	59.53±12.41	21.47±7.01	AROM 82.60±16.70 PROM 89.53±17.44	AROM 111.93±22.32 PROM 117.20±21.97	
Chauhan et al., 2011 18	26 participants (11 male & 15 female) Age=40-60 years	Experimental	DTFM and conventional physical therapy, 3 days a week for 2 weeks	NR	NR	NR	NR	The study reported that improvements in shoulder ROM and pain were significantly (P <0.05) better in the experimental group post treatment.
		Control	Conventional physical therapy, 3 days a week for 2 weeks	NR	NR	NR	NR	
(Vijayan & Jayabharathi. 2019 19	30 male and female participants Age=40-60 years	Experimental	DTFM with mobilization technique, 15 minutes session, 5 days per week for 3 weeks	58.67±11.68	48.71±10.28	ABD 50.33±13.16 ER 15.67±7.53 FLX 98.33±19.97	73.21±14.09 23.93±8.59 111.79 ±21.89	In comparison to control group, ROM was significantly (P <0.05) improved in experimental group. Nonetheless there was no significant difference (P>0.05) between the groups regarding pain.
		Control	Muscle energy technique with mobilisation technique, 1 session per day, 5 days per week for 2 weeks	54.13±10.97	43.71±10.78	ABD 44.33±15.45 ER 12.44±5.94 FLX 97.33±18.98	86.07±19.73 33.57±8.42 118.21±15.14	

ABD, Abduction; AROM, Active Range of Motion; DTFM, Deep transverse friction massage; ER, External Rotation; FLX, Flexion, IR, Internal Rotation; NR, Not Reported; PNF, Proprioceptive Neuromuscular Facilitation; ROM, Range of Motion.

**Table-III:** Methodological quality of the included studies.

CASP Score	Sonkusale et al.	Guler-Uysal & Kozandoglu,	Mogahed et al.	Sah et al.	Chauhan et al.	Vijayan & Jayabharthi
Was the trial’s topic fully defined?	Y	Y	Y	Y	Y	Y
Was the trial’s topic fully defined?	Y	Y	Y	Can’t tell	Can’t tell	Y
At the trial’s conclusion, were all of the participants accurately counted?	Can’t tell	Can’t tell	Can’t tell	Can’t tell	Can’t tell	Can’t tell
Were all the individual’s blind?	N	N	N	N	N	Can’t tell
Were the groups comparable in the beginning?	Y	Y	Y	Y	Y	Y
Were the various groups handled fairly?	Y	Y	Y	Y	Y	Y
How significant was the therapeutic impact?	Y	Y	Y	Y	N	Y
How accurate was the treatment effect estimate?	N	N	N	N	N	N
Can the findings be applied to the community	Y	Y	Y	Y	Y	Y
Were all clinically significant outcomes taken into account?	Y	Y	Y	Y	Y	Y
Were all clinically significant outcomes taken into account?	Y	Y	Y	Y	Y	Y

Total score	8	8	8	7	6	8

Clinical trials published in English language from the earliest record to March 2022 that reported effects of DTFM/Cyriax’s friction massage on pain and/or ROM in patients with diagnosis of adhesive capsulitis were included. Reviews, commentaries, letter to editors and conference papers were excluded.

Search results from different databases and additional searching were imported into Rayyan (www.rayyan.ai) to remove duplicates and perform screening. Two authors (SK and SA) independently screened the titles and abstracts of the studies and divided the articles into “include” “exclude” and “unsure” categories. Full text screening was performed by the same authors (SK and SA), who performed the title and abstract screening. Discrepancies between the two authors (SK and SA) were resolved by consensus meeting, and if needed, third reviewer (AA) was consulted to make the final decision. A data extraction form was designed in MS excel to extract data from the included studies. The data extraction form was piloted before the data extraction.

The Critical Appraisal Skills Programme (CASP) was used for quality assessment of the included studies. CASP is a well-structured, widely used quality appraisal tool. The CASP checklist comprises of eleven questions divided into three categories (A, B, and C). The first three questions act as screening questions, establishing whether it is appropriate to continue with the remaining questions or not. The remaining questions are more in-depth questions on particular areas of the paper’s methodology. Each question in a CASP checklist for RCT is given one point, so the CASP checklist has a total score of eleven. Studies with a score of 7/11 or higher are regarded to be of good quality. Studies with a score of four to six out of 11 are considered medium quality, while those with a score of less than four are called low quality studies.^14^,^15^

## RESULTS

**Fig.1:**
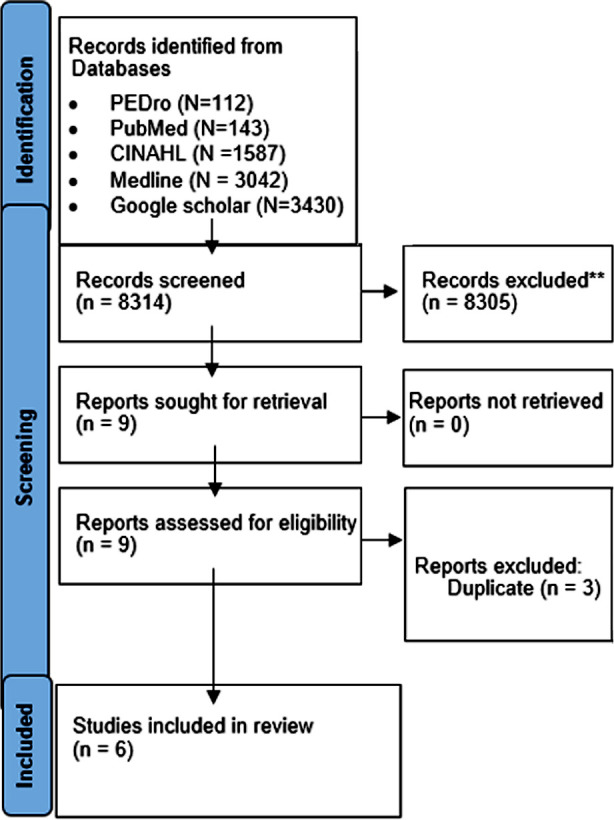
PRISMA Flow chart (Studies selection process).

Initial search identified 8,314 research articles. Titles and abstracts of these articles were screened, and 8,305 studies were excluded because these were irrelevant. Of the remaining nine studies, three studies were excluded at full text screening stage. Finally, six publications reporting on 226 patients were included in the systematic review (Fig.1).

The six studies that were selected for this systematic review were all randomized controlled trials. Three studies^16^-^18^ made the diagnosis of adhesive capsulitis based on (i) History of pain lasting equal to or less than two months, (ii)Capsular pattern, (iii) Loss of ROM both active and passive by more than 50% in comparison to the sound side, (iv) Shoulder radiographs and (v) No other shoulder or medical conditions. Sonkusale et al. kept the same criteria but included only idiopathic adhesive capsulitis patients.^12^ Vijayan & Jayabharathi S recruited individuals with clinically diagnosed adhesive capsulitis (Stage-II), with a minimum duration of two months and a marked decrease of passive and active ROM.^19^ Mogahed et al., included only female post-mastectomy adhesive capsulitis patients who had scapulo-thoracic and glenohumeral changes but no other shoulder disorders.^20^

Four studies reported that pain was significantly (P<0.05) improved in experimental group as compared to control group.^12^,^16^,^18^,^20^ Only two studies showed non-significant differences.^17^,^19^ Five studies^12^,^16^,^18^-^20^ showed that ROM was significantly (P<0.05) improved in experimental group as compared to control group while only one study^17^ reported non-significant (P=0.107) results.

On the CASP tool, four studies had a score of 8/11^12^,^16^,^19^,^20^, while the other two studies received a score of 7/11^17^ and 6/11.^18^ One of the study^18^ included in this review were of medium quality while five studies^12^,^16^,^17^,^19^,^20^ had high quality.

## DISCUSSION

The study aim was to assess the effectiveness of DTFM in treating patients with adhesive capsulitis. Pain is a common symptom of adhesive capsulitis and the primary outcome in all the included studies in current review was pain. The findings of the review demonstrated that patients in the DTFM groups experienced significant pain reduction as compared to control group. Only two of the included studies contradict the effectiveness of DTFM as compared to control group.

Out of the total six studies, five studies applied the DTFM for two weeks. Only one study had a treatment period of three weeks.^19^ Studies that have been conducted on the problem of recovery from adhesive capsulitis have found that the length of the recovery period can be noticed in individuals anywhere from one to four years following the initial start of the symptoms.^21^ There is a widespread agreement among medical professionals that non operative care is the most effective form of first line of treatment for adhesive capsulitis.^22^ The long-term prognosis of adhesive capsulitis cannot be changed by non-operative therapies, although they do bring symptomatic pain relief and enhance shoulder range of motion in the short term.^23^ Evidence suggests that the healing of adhesive capsulitis will occur as expected given sufficient time, which can take from around two to three years on average.^23^-^25^ Therefore, studies that examine DFTM over a longer timeframe are needed because the included studies in current review assessed the short-term outcomes of DTFM.

The pain relief produced by DFTM may be the result of the control of nociceptive impulses at the level of the spinal cord.^16^,^26^,^27^ According to Cyriax, friction causes an increase in the breakdown of pain-inducing metabolites such as Lewis’ chemicals. Another mechanism that may help in reducing pain is diffuse noxious inhibitory control, which is a pain suppression mechanism that produces endogenous opiates, reducing the integrity of pain communicated to higher centers.^28^-^30^

The ROM was the second outcome of the review. The results of the included studies showed that the outcome regarding the ROM significantly improved in patients who receive DTFM. Only one study reported that control group had the same efficacy as the DTFM intervention in improving ROM.^17^ To increase ROM, adhesions may be broken down and collagen may be realigned because of mechanical changes that take place when certain movements put pressure on particular regions of the capsular tissue.^31^ Deep friction massage applied in a transverse direction moved the tendon to and pro, which freed the tissue from adhesions that were already present as well as those that were still in the process of forming. The transverse movement reproduces the normal mobility of the structure by widening the interfibrillary adhesions without stretching them. This keeps them from adhering together and gives the structure a wider range of motion.^27^

The studies included in the review had varying sample sizes. The study conducted by Chauhan et al.^18^ had a small sample size of 26, whereas the average sample size for the other included studies was 37, with Sonkusale et al.^12^ study having the largest sample size of 60. The sample size of the study has an impact on generalizability. A large sample size helps clinicians to trust and generalize study results.^32^,^33^ The internal validity of the included papers in the review were appraised for their methodological qualities using the CASP tool and the findings showed that majority of the studies had high quality.

### Limitations:

The review only considered studies that had already been published, which might have created publication bias. It’s possible that the author missed some important data from unpublished studies, which might have changed the review conclusion. Furthermore, due to substantial heterogeneity in the included studies, we were unable to conduct a meta-analysis. It could not be registered with PROSPERO because when it was conducted, they were registering just COVID19 pandemic related protocols and there is no option for retrospective registration with them.

## CONCLUSION

Considering the review overall findings, it can be concluded that DTFM significantly relieve pain and improve ROM both immediately and in short term in individuals with adhesive capsulitis. Large, multicenter randomized controlled trials with longer follow-ups are recommended to determine the long-term effectiveness of DTFM in managing adhesive capsulitis patients.

### Authors’ Contribution:

**SK:** Concept and study design, literature search, data extraction, drafting the manuscript, final approval of the version to be published.

**AA:** Concept and study design, data extraction, critical revision, final approval of the version to be published and responsible for the integrity and accuracy of the manuscript.

**SK:** Literature search, drafting the manuscript, final approval of the version to be published.

**SA:** Literature search, data extraction, critical revision, final approval of the version to be published.
